# SdiA, an *N*-Acylhomoserine Lactone Receptor, Becomes Active during the Transit of *Salmonella enterica* through the Gastrointestinal Tract of Turtles

**DOI:** 10.1371/journal.pone.0002826

**Published:** 2008-07-30

**Authors:** Jenee N. Smith, Jessica L. Dyszel, Jitesh A. Soares, Craig D. Ellermeier, Craig Altier, Sara D. Lawhon, L. Garry Adams, Vjollca Konjufca, Roy Curtiss, James M. Slauch, Brian M. M. Ahmer

**Affiliations:** 1 Department of Microbiology, The Ohio State University, Columbus, Ohio, United States of America; 2 Department of Microbiology and College of Medicine, University of Illinois, Urbana, Illinois, United States of America; 3 Department of Population Health and Pathobiology, North Carolina State University, Raleigh, North Carolina, United States of America; 4 Department of Veterinary Pathobiology, College of Veterinary Medicine, Texas A&M University, College Station, Texas, United States of America; 5 Department of Biology, Washington University, St. Louis, Missouri, United States of America; Massachusetts General Hospital, United States of America

## Abstract

**Background:**

LuxR-type transcription factors are typically used by bacteria to determine the population density of their own species by detecting N-acylhomoserine lactones (AHLs). However, while *Escherichia* and *Salmonella* encode a LuxR-type AHL receptor, SdiA, they cannot synthesize AHLs. In vitro, it is known that SdiA can detect AHLs produced by other bacterial species.

**Methodology/Principal Findings:**

In this report, we tested the hypothesis that SdiA detects the AHL-production of other bacterial species within the animal host. SdiA did not detect AHLs during the transit of *Salmonella* through the gastrointestinal tract of a guinea pig, a rabbit, a cow, 5 mice, 6 pigs, or 12 chickens. However, SdiA was activated during the transit of *Salmonella* through turtles. All turtles examined were colonized by the AHL-producing species *Aeromonas hydrophila*.

**Conclusions/Significance:**

We conclude that the normal gastrointestinal microbiota of most animal species do not produce AHLs of the correct type, in an appropriate location, or in sufficient quantities to activate SdiA. However, the results obtained with turtles represent the first demonstration of SdiA activity in animals.

## Introduction

The functions of more than half of the genes present in *Escherichia coli* and *Salmonella enterica* serovar Typhimurium (hereafter referred to as *S.* Typhimurium) have never been determined, despite decades of genetic screens and selections. This failure is hypothesized to result from a lack of gene expression or gene function in the laboratory environment. A major component of the natural environment missing from most laboratory experiments is the presence of other microbial species. *E. coli* and *S.* Typhimurium colonize and propagate in the intestines of animals in the presence of large numbers of other microbial species. We have previously identified genes of *S.* Typhimurium that are expressed specifically in the presence of signaling molecules produced by other microbes [1]–[4]. These seven genes (two loci) are activated by SdiA, a member of the LuxR family of transcription factors, in response to *N*-acylhomoserine lactones (AHLs) produced by other species.

The use of LuxR homologs to detect the AHL production of other species is unusual. The typical function of LuxR homologs is “quorum sensing”, a phenomenon by which bacteria sense their own population density [5]–[7]. Many Gram-negative bacteria use this information to regulate the production of host colonization factors. For instance, *Vibrio fischeri* uses this information to regulate genes that play a role in colonization of the squid, *Euprymna scolopes* [8]–[10]. Numerous plant and animal pathogens use quorum sensing to regulate host interaction genes [11], [12]. Presumably, this prevents the bacteria from alerting host immune responses before a population sufficient to overcome host defenses has been assembled.

AHLs are synthesized using enzymes of the LuxI or LuxM family [13]–[18]. AHLs vary widely in the length of their acyl chain (from four to 18 carbons) and can be modified at the 3-carbon position to have a carbonyl or hydroxyl group. Each LuxI homolog produces predominantly a single AHL variant, along with smaller quantities of closely related variants [19]. The LuxR family member of a species can detect nanomolar concentrations of the AHL produced by that species, providing some species specificity to the system. However, LuxR homologs can often detect other related AHLs with lower sensitivity [20].

*Salmonella* encodes a LuxR homolog, SdiA, but does not encode an AHL synthase [3], [4]. Instead, SdiA responds to AHLs produced by other microbial species [1], [2]. SdiA detects a much wider range of AHLs than other LuxR homologs, although with varying sensitivities. The most potent AHL is 3-oxo-octanoyl-homoserine lactone (oxoC8) and SdiA responds to this molecule at concentrations of 1 nM or higher [3], [21]. The closely related AHL, 3-oxo-hexanoyl-homoserine lactone (oxoC6) can be detected at concentrations above 5 nM, and at 50 nM SdiA responds to oxoC4, oxoC10, oxoC12, and the unmodified AHLs C6 and C8. At concentrations above 1 µM SdiA responds to C4, C10, and C12 [3], [21]. The three-dimensional structure of the N-terminus of *E. coli* SdiA bound to C8 has been determined using NMR, confirming that detection of AHLs by SdiA is direct [22].

Upon detection of AHL, SdiA activates the expression of two *srg* (SdiA regulated gene) loci: the *rck* operon located on the *S.* Typhimurium virulence plasmid, pSLT, and the *srgE* gene located in the chromosome at 33.6 centisomes [1], [2]. The *rck* operon is directly downstream of the *pef* operon that encodes plasmid-encoded fimbriae [23]. These fimbriae play a role in adhesion to the small intestine of mice [24]. The *rck* operon contains six genes: *pefI*, *srgD*, *srgA*, *srgB*, *rck*, and *srgC* [3], [23]. PefI is a regulator of the upstream *pef* operon [25]. SrgD is a putative transcription factor with a helix-turn-helix motif of the LuxR family, but its target genes have not been identified [3], [23]. SrgA is a DsbA homolog that catalyzes disulfide bond formation of the Pef fimbrial subunit and other periplasmic proteins [26], [27]. SrgB is a lipoprotein of unknown function [23]. Rck is an 8-stranded β-barrel protein localized to the outer membrane [28]. This protein has dual functions. The first function provided its name: resistance to complement killing [29], [30]. More specifically, Rck prevents the polymerization of complement component C9 on the bacterial surface [31]. Second, Rck serves as an adhesin to fibronectin and laminin [32]. The last gene in the *rck* operon, *srgC*, is a regulator of the AraC-family of transcription factors whose target genes are unknown [23]. The second *srg* locus consists of a single gene, *srgE*, which is predicted to encode a protein containing a coiled-coil domain [2], [4]. As with the *rck* operon, *srgE* is not present in *E. coli* and was probably acquired horizontally by *Salmonella*. Despite the regulation of putative virulence genes by SdiA, we have previously reported that an *sdiA* mutant of *S.* Typhimurium is not attenuated in mouse, chicken, or cow models of infection [1].

The mammalian intestinal community is comprised of approximately 800 microbial species [33]. We hypothesized that members of this community utilize AHL-type quorum sensing and that the presence of these AHLs would provide an effective way for *S.* Typhimurium to detect the intestinal environment [1], [4]. In this report we have tested this hypothesis. *S.* Typhimurium did not detect AHLs during transit through the intestinal tract of several species of animals, indicating that the normal microbiota of these hosts did not produce AHLs of the correct type or at a sufficient concentration to activate SdiA. However, SdiA was active during transit through turtles colonized by the AHL-producing organism, *Aeromonas hydrophila*.

## Results

### Construction and testing of RIVET strains

To test the hypothesis that *Salmonella* uses SdiA to detect the AHL production of other microorganisms within the gastrointestinal tract, we used the RIVET method (Recombination-based In Vivo Expression Technology) to record SdiA activity in vivo [34]–[37]. We constructed a *srgE*-*tnpR*-*lacZY* transcriptional fusion in a strain that carries a *res1*-*tetRA*-*res1* cassette elsewhere in the chromosome (strains are listed in Table 1). The *tnpR-lacZY* fusion was placed immediately after the *srgE* stop codon so that *srgE* remains functional. The *tnpR* gene encodes a resolvase that catalyzes a site-specific recombination event at *res1* target sequences. Thus any condition or event that induces expression of *srgE*-*tnpR-lacZY* is permanently recorded in the genome by the deletion of the *tetRA* cassette. The deletion event is detected by screening the bacteria for a loss of tetracycline resistance.

**Table 1:** Strains and plasmids used.

Strain or plasmid	Genotype	Source or reference
**Strains**		
14028	Wild type *Salmonella enterica* serovar Typhimurium	American Type Culture Collection
BA612	14028 *sdiA1*::mTn*3* (amp^r^)	[4]
JNS3206	JS246 *srgE10-tnpR-lacZY* (kan^r^)	This study
JNS3226	JS246 *srgE10-tnpR-lacZY sdiA1*::mTn*3* (kan^r^ amp^r^)	This study
JS198	LT2 *metE551 metA22 ilv452 trpB2 hisC527*(am) *galE496 xyl-404 rpsL120 flaA66 hsdL6 hsdSA29* zjg8103::*pir* ^+^ *recA1*	[56]
JS246	14028 zjg8103::*res1-tetRA-res1*	[37]
S17-1λpir	*E. coli recA pro hsdR* <RP4-2-*tet*::Mu-1*kan*::Tn*7*> λpir	[60]
W3110	*E. coli* K-12 F^−^ IN(*rrnD-rrnE*)*1*	[61]
**Plasmids**		
pCE71	FRT-*tnpR-lacZY* t*_his_* oriR6K (kan^r^). Contains wild type *tnpR* Shine Dalgarno. FRT orientation B.	[37]
pCE73	FRT-*tnpR-lacZY* t*_his_* oriR6K (kan^r^). Contains mutated *tnpR* Shine Dalgarno *mut168*. FRT orientation B.	[37]
pCE75	FRT-*tnpR-lacZY* t*_his_* oriR6K (kan^r^). Contains mutated *tnpR* Shine Dalgarno *mut135*. FRT orientation B.	[37]
pCP20	*cI857* λP_R_ *flp* pSC101 oriTS (amp^r^ cam^r^)	[58]
pJNS25	P_srgE_-*luxCDABE* p15A ori (tet^r^)	[2]
pKD4	FRT-*kan*-FRT oriR6K (amp^r^)	[57]
pKD46	P_BAD_ *gam bet exo* pSC101 oriTS (amp^r^)	[57]

To obtain the optimal fusion sensitivity for the *srgE* promoter three versions of this strain were constructed, each with a different *tnpR* ribosome binding site [38]. Once these strains were constructed, an *sdiA1*::mTn*3* mutation was transduced into each of them using phage P22HTint to create isogenic *sdiA* mutant controls. We have previously reported that even in the presence of AHLs, SdiA is not active when *Salmonella* is grown on LB agar plates [2]. However, SdiA is slightly active when grown in liquid broth, and is highly active when grown in motility agar [2]. Therefore, the functionality of the RIVET strains was tested under these same conditions. Either synthetic AHL (1 µM oxoC6) or the solvent control, acidified ethyl acetate (EA), was added to each medium. After overnight growth in liquid medium at 37°C, serial dilutions were plated on agar plates to obtain individual colonies. Bacteria were recovered from motility agar by stabbing the agar with a sterile toothpick and streaking to isolation. The resulting colonies were then screened for loss of tetracycline resistance. The strains containing mutated *tnpR* ribosome binding sites showed no resolution under any condition and were not used further. The strains containing the wild-type *tnpR* ribosome binding site showed resolution and are the strains used for the remainder of the experiments in this report (JNS3206 is *sdiA* ^+^ and JNS3226 is *sdiA*::mTn*3*, hereafter referred to as the RIVET strains). In media containing AHL, resolution was observed in 23% of wild-type bacteria recovered from motility agar, 2.5% of wild-type bacteria recovered from broth, and from 0.6% of bacteria grown on solid agar. No resolution was observed under any growth condition in the absence of AHL or from *sdiA* mutant colonies. The reason that only 23% of colonies resolve after overnight growth in vitro is not known, but indicates that the *srgE* gene is not highly expressed, or that truly optimal conditions have not yet been discovered. Regardless, resolution in the *sdiA* ^+^ bacteria but not the *sdiA* mutant bacteria indicates SdiA activity. The observation that the highest resolution was observed after growth in motility agar is similar to what was observed previously using a *lacZY* fusion to *srgE* [2]. Therefore, expression of the *srgE*-*tnpR-lacZY* fusion can be recorded as a percentage of tetracycline susceptible colonies and this expression is dependent upon *sdiA* and AHL.

### SdiA is not active during the transit of *Salmonella* through mice, chickens, pigs, a rabbit, a guinea pig, or a cow

**Figure 1:**
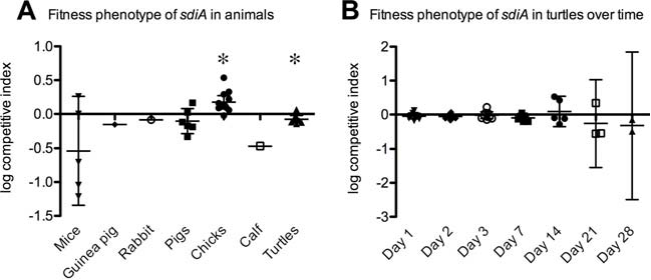
Fitness of the *sdiA* mutant in different animal species. (A) The log of the competitive index for each individual animal is plotted. The mean is indicated by the large horizontal line while the 95% confidence intervals are indicated by the smaller horizontal lines. A value of zero indicates that the wild-type and the *sdiA* mutant were of equal fitness during transit through the animal. (*) The wild-type was statistically more fit than the *sdiA* mutant during transit through turtles, while the *sdiA* mutant was statistically more fit than the wild-type during transit through chicks (p<0.05). (B) Fitness of the *sdiA* mutant in turtles over time. The data from (A) is separated into time points. (*) The wild-type was statistically more fit than the sdiA mutant on day 7 (p<0.05).

To determine if SdiA is active during the transit of *Salmonella* through the gastrointestinal tract, the wild-type and *sdiA* mutant RIVET strains were mixed together in a 1∶1 ratio and orally administered to six CBA/J mice. These mice are resistant to *Salmonella* infection and provide a long term model of intestinal colonization and persistence [39]. Inoculating a mixture of the wild-type and *sdiA* mutant allows a comparison of the resolvase activity of these two strains within the gut of the same animal. Fecal samples were collected, homogenized and plated on XLD indicator plates containing kanamycin to recover the *Salmonella* RIVET strains. These colonies were confirmed to be *Salmonella* by their black color on the XLD indicator plates and their sensitivity to phage P22. No *Salmonella* were recovered from the feces of uninfected mice. Each recovered colony was then screened for ampicillin and tetracycline resistance. Wild-type and *sdiA* mutant are distinguished by the ampicillin sensitivity of the colony (the *sdiA1*::mTn*3* mutation encodes ampicillin resistance) and resolvase activity is detected by the loss of tetracycline resistance. Sufficient numbers of bacteria for screening were recovered from five of the six mice. Only one of 717 colonies recovered from the five mice was tetracycline susceptible, indicating that SdiA was not active during transit through the mice (Table 2). Consistent with the lack of SdiA activity, both the wild-type and *sdiA* mutant were recovered in ratios that were not significantly different from the ratio inoculated indicating that the *sdiA* mutation did not confer a fitness phenotype in these mice (Table 2 and Figure 1).

**Table 2:** SdiA activity and fitness during transit through different animal species.

Number and type of animal^1^	Percent wild-type inoculated^2^	Total colonies screened^3^	Percent wild-type recovered^3^	Competitive Index^4^	Mean of the log competitive index+/−Std Dev^4^	Total colonies resolved^3^	Percent of wild-types resolved	Percent of *sdiA* mutants resolved
5 mice	52%	717	72%	0.41	−0.541+/−0.645	1	0.14%	0%
1 guinea pig	45%	621	54%	0.70	−0.155	0	0%	0%
1 rabbit	45%	709	50%	0.82	−0.086	0	0%	0%
6 pigs^5^	45%	1349	53%	0.73	−0.105+/−0.176	0	0%	0%
1 calf^6^	89%	1000	96%	0.34	−0.469	0	0%	0%
12 chicks^7^	57%	2454	48%	1.44	0.176+/−0.150*	0	0%	0%
8 turtles^8^	49%	6793	56%	0.75	−0.078+/−0.068*	715	25.9%	3.0%

1 Animals were inoculated with a 1∶1 mixture of the wild-type and *sdiA* mutant *Salmonella* RIVET strains (a total of 9.7×10^8^ cfu for the mice, 6.0×10^9^ cfu for the guinea pigs and rabbits, 1.8×10^9^ cfu for the pigs, 1.3×10^11^ cfu for the calves, 7.0×10^9^ cfu for the chicks, and 3.1×10^9^ cfu for turtles).

2 The inoculum was plated for single colonies and screened for ampicillin resistance to determine the actual percent wild-type inoculated.

3 The “Total colonies screened” represents a compilation of all colonies recovered (sum of all time points) from all animals of each species. The colonies were recovered by plating fecal samples, unless noted otherwise, on XLD-kan and then screened for ampicillin and tetracycline resistance. Wild-type and *sdiA* mutant *Salmonella* are differentiated by ampicillin resistance and resolution of the *res1-tetRA-res1* cassette is indicated by tetracycline sensitivity.

4 The competitive index equals the output ratio (cfu of mutant/cfu of wild type) divided by the input ratio (cfu of mutant/cfu of wild type). A competitive index of 1.0 indicates that the wild-type and *sdiA* mutant had equal fitness during transit through the animal. The log of the competitive index represents a normal distribution so the mean of this value was calculated. A value of zero indicates that the wild-type and *sdiA* mutant had equal fitness during transit through the animal. For those animal species in which colonies were recovered from more than one animal, the standard deviation is shown. The wild-type was statistically more fit than the *sdiA* mutant in turtles, while the *sdiA* mutant was more fit than wild-type in chicks (student's t test, p<0.05). However, the differences are so small (competitive index of less than 3) that they are not likely to be biologically significant. For those animal species where only one individual shed colonies, the standard deviation could not be calculated, but a competitive index that is less than 3-fold different than 1.0 is unlikely to be biologically significant (0.33 to 3.0).

5 Of the total screened, 700 colonies were from fecal samples, 582 were from the ileum, and 67 were from the cecum.

6 Of the total screened, 782 colonies were from fecal samples, 150 were from the ileum, 2 were from the cecum, and 66 were from the Peyer's patches.

7 All colonies from the chicks were obtained from the cecum and distal large intestine. No colonies were from fecal samples.

8 All colonies from the turtles were obtained from cloacal swabs. These data were also used to generate Figures 1 and 3.

* Statistically significant fitness difference between wild-type and *sdiA* mutant (p<0.05).

The lack of SdiA activity in the mouse gut was surprising, but since compounds that activate AHL biosensor strains have been chemically extracted from the bovine rumen [40], we hypothesized that the presence of AHLs might differ among animal species. Therefore, the RIVET strains were orally administered to cows, pigs, rabbits, guinea pigs, and chickens, as described in the Materials and Methods. For some individual animals, we failed to recover the RIVET strains from their feces. However, the RIVET strains were recovered from the feces of one rabbit (of two), one guinea pig (of four), one cow (of three), six pigs (of six), and 12 chickens (of 12). No SdiA activity was observed during the transit of *Salmonella* through any of these animals as indicated by the fact all recovered RIVET strains were tetracycline resistant (Table 2). Consistent with the lack of SdiA activity, the *sdiA* mutant did not appear to have a significant fitness defect during the transit through any of the animals (Table 2 and Figure 1). It should be noted that the *sdiA* mutant actually faired slightly better than the wild-type during transit through chicks. The *sdiA* ^+^:*sdiA* ^−^ ratio was 57∶43 at inoculation while it was 48∶52 at recovery. This is a competitive index of 1.4. While this result was statistically significant, competitive indices of even 3 to 5 are considered minimal.

### SdiA is active during the transit of *Salmonella* through turtles

**Figure 2:**
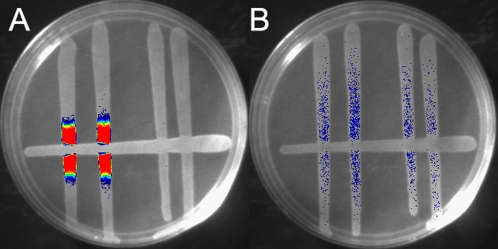
Cross-streak assay demonstrates that the S. Typhimurium *srgE* gene is activated in an *sdiA*-dependent fashion in the vicinity of *Aeromonas hydrophila.* (A) A turtle isolate of *Aeromonas hydrophila* was struck across the bottom of an LB agar plate. On the left side, a wild-type S. Typhimurium strain carrying a *srgE-luxCDABE* fusion (14028/pJNS25) was struck in duplicate and perpendicular to the *Aeromonas.* On the right side, an isogenic *sdiA* mutant was struck in the same fashion (BA612/pJNS25). Light intensity is pseudocolored with blue being the weakest and red being the most intense. (B) Same as panel A except that an AHL-negative organism was struck across the bottom of the plate (*E. coli* K-12 strain W3110).

Having determined that *Salmonella* did not detect AHLs in the gastrointestinal tracts of the birds and mammals tested above, we decided to test a reptile that is commonly associated with *Salmonella*, the turtle. Preliminary experiments indicated that SdiA was indeed activated during transit through turtles (not shown). To determine if AHL-producing bacteria could be recovered from turtles, cloacal swab samples were obtained from a group of eight turtles and the recovered material was plated on several media in order to culture a wide variety of bacterial species (LB, blood agar, XLD, lactose MacConkey). The recovered colonies were then tested in a cross-streak assay against the bioluminescent SdiA activity reporter strains 14028/pJNS25 (*sdiA* ^+^ *srgE-luxCDABE*) and BA612/pJNS25 (*sdiA* ^−^ *srgE-luxCDABE*) [2]. Luminescence of the wild-type reporter but not the *sdiA* mutant at the junction indicates SdiA activity [2]. Bacteria that can activate SdiA in the cross-streak assay were found in each of the eight turtles (Figure 2). Eighteen isolates were submitted to the Ohio State University medical center for automated identification by a Dade Microscan WalkAway 96si. All 18 isolates were found to be *Aeromonas hydrophila*.

**Figure 3:**
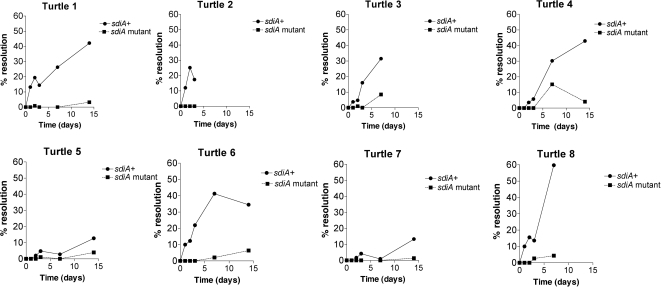
Resolution over time in eight turtles that were culture positive for *Aeromonas hydrophila.* A 1∶1 mixture of the *sdiA+* and *sdiA::mTn3* RIVET strains (JNS3206 and JNS3226, respectively) were orally administered to the eight turtles (3.1×109 total cfu). Plating of cloacal swabs was used to recover the strains at time points. Each colony recovered was then screened for ampicillin resistance to determine if it is *sdiA+* or *sdiA::mTn3*, and tetracycline resistance to determine if it had resolved. The percentage of *sdiA+* (•) and *sdiA::mTn3* (▪) resolution is plotted over time. Turtles 2, 3, and 8 were euthanized before the final time point.

After determining that all eight of the turtles contained *Aeromonas hydrophila*, the *Salmonella* RIVET strains were orally administered to these turtles and cloacal swab samples were collected over time. When the recovered colonies from all turtles at all time points were screened, it was observed that the ratio of wild-type to *sdiA* mutant recovered was changed during transit through the turtles (a 49∶51 ratio inoculated, a 56∶44 ratio recovered, Table 2 and Figure 1). However, while statistically significant, this is only a 1.3-fold difference in competitive index that is not likely to be biologically relevant, especially when looking at the time course plotted in Figure 1B. Therefore, we do not feel that the *sdiA* mutant of *Salmonella* serovar Typhimurium has a significant fitness phenotype in turtles. However, the striking result is that the resolution observed in wild-type *Salmonella* was greater than in the *sdiA* mutant, indicating that SdiA was active during the transit of *Salmonella* through these turtles (Table 2 and Figure 3). This is the first observation of SdiA activity in vivo.

To test the hypothesis that *Aeromonas hydrophila* is indeed the organism responsible for activation of SdiA during the transit of *Salmonella* through turtles, we hoped to obtain a group of turtles that lacked AHL-producing bacteria. Half of these turtles would then be inoculated with *Aeromonas hydrophila* and half would be inoculated with LB prior to performing the RIVET experiment. Unfortunately, the next two groups of turtles that we obtained (one group of 12 and another group of 17) all had *Aeromonas* in their feces. Next, we reasoned that newly hatched turtles would be less likely to have picked up *Aeromonas* from the environment and we would have a better chance of obtaining *Aeromonas*-free animals if we ordered hatchlings. A group of 20 hatchlings was obtained and, unfortunately, all of these animals were colonized with *Aeromonas*. In a final attempt at obtaining *Aeromonas*-free turtles, we ordered 30 turtle eggs and hatched them under sterile conditions. More specifically, we placed the eggs on sterile vermiculite until hatching, and then placed them in sterile cages containing sterile water. The hatchlings were fed standard non-sterile turtle food to promote the development of a normal microbiota (the food was tested and shown to be free of *Aeromonas*). Remarkably, all of the hatchlings were found to have *Aeromonas* in their feces. We are not sure if the *Aeromonas* is transmitted from the mother to the embryo during the development of the egg, or if there was *Aeromonas* on the egg shell, or some other source of environmental contamination, but we were not able to obtain turtles that lacked *Aeromonas*.

Next, we attempted to clear the *Aeromonas* from adult turtles using Amikacin as described in the Materials and Methods. This was not successful. *Aeromonas* numbers would drop temporarily but the organism was never completely eradicated. Therefore, we can only conclude that SdiA was indeed activated during the transit of *S.* Typhimurium through turtles, and that the probable source of the AHLs was *Aeromonas hydrophila*.

## Discussion

We have determined that SdiA of *S.* Typhimurium is activated in turtles, but not in mice, pigs, chickens, a cow, a guinea pig, and a rabbit. It appears that the normal microbiota of these particular animals did not produce AHLs, at least not in a location or at a concentration sufficient to activate SdiA. The lack of SdiA activity during the transit of *Salmonella* through the cow was particularly surprising because there is a report of chemical extraction of AHLs from the rumens of six of eight bovines [40]. It is certainly possible that AHLs are a normal component of the bovine rumen and certain other gastrointestinal regions of particular animals. We may not have observed SdiA activity in our bovine experiment (or other experiments) due to the age or the diet of the animals. Our calves were young, only three to four months old. While the animals have a functioning rumen at this point, AHL-producing species may not be part of the ecosystem at this age. The ages of the animals in the Erickson study were not reported. Diet may also play a role. Our calves were fed free choice grass and hay and a pelleted feed that was 12% protein and 3% fat that is primarily comprised of corn and cottonseed meal. The Erickson study used four different combinations of alfalfa hay and barley, but did not observe a statistically significant correlation between diet and AHLs. Further study will be required to determine the circumstances in which AHL production occurs in the bovine rumen.

It is possible that AHLs were indeed synthesized by the normal microbiota of some of the animals in this study, but that the AHLs were rapidly degraded by either host enzymes or enzymes produced by other microbes. While AHL-degrading enzymes have not been reported in the intestine, they have been found in soil and in the termite hindgut, and it would not be surprising to find them in the intestine [41]–[43]. While enzymatic degradation may occur, the *Salmonella* SdiA activity observed in turtles infected with *Aeromonas* argues that AHLs can survive the turtle intestinal environment in a concentration sufficient to activate SdiA. It will be interesting to determine the half-life of AHLs in the intestines of various animal species and to determine the distances over which signaling can occur.

The SdiA activity observed during the transit of *Salmonella* through turtles was likely due to the AHL production of *Aeromonas hydrophila*. This organism is a major fish pathogen that causes septicemia and Ulcerative Disease Syndrome and is among the most troublesome of bacterial pathogens in aquaculture [44]–[46]. It also causes gastroenteritis, peritonitis, and meningitis in humans [47]. Many of the *Aeromonas* virulence factors are regulated by the AhyR/AhyI quorum sensing system in which AhyI is an AHL synthase. When a high population density is achieved at a focus of infection, a positive feedback loop is initiated in which AhyR activates the *ahyI* gene, thus making more AHL signaling molecules to activate AhyR. In this way, the population of organisms can simultaneously express virulence factors via AhyR and attack the host [48]–[50].

The activation of the *srgE-tnpR* fusion during the transit of *Salmonella* through turtles was dependent on the *sdiA* gene. This is consistent with in vitro cross-streak assays in which *Salmonella* can detect *Aeromonas* in an *sdiA*-dependent fashion (Figure 2). However, the fitness phenotype in turtles was extremely small. When a 49∶51 mixture of *sdiA* ^+^ and *sdiA* ^−^ RIVET strains was given to turtles, a 56∶44 mixture was recovered in the feces indicating that the *sdiA* mutant faired slightly worse than the wild-type in this environment. This is a competitive index of 0.75 which is very close to 1.0. While this result was statistically significant, it is not considered significant in the field of bacterial pathogenesis where even 3 to 5-fold differences in competitive indices are considered minimal. Given that *S.* Typhimurium is not a serovar that is typically associated with turtles, *S.* Typhimurium may not have *sdiA*-regulated genes that play a role in this animal. It would be interesting to determine if *sdiA* mutants of turtle-associated serovars such as Muenchen, Arizonae, or Newport [51]–[53] have a phenotype in *Aeromonas*-infected turtles. While not typically associated with turtles, *S.* Typhimurium is an exceptionally broad host-range pathogen, causing gastroenteritis in humans, cattle, pigs, poultry, horses, and sheep. It is quite common in humans, accounting for 26% of all U.S. isolates [54]. It will be interesting to determine if serovar Typhimurium detects AHL-producing pathogens in any of these other hosts and if *sdiA* confers a fitness advantage in any of these coinfection situations.

## Materials and Methods

### Bacterial strains and media

All bacterial strains and plasmids used are listed in Table 1. *E. coli*, *Salmonella*, and *Aeromonas* were grown in Luria-Bertani (LB) broth at 37°C unless otherwise indicated (EMD chemicals, Gibbstown NJ). LB agar plates and LB motility agar were made by adding agar to 1.2% or 0.25%, respectively (EMD chemicals). For recovery of *Salmonella* and *Aeromonas* from animal tissues or feces, xylose-lysine-deoxycholate (XLD) agar plates were used (EMD chemicals). The antibiotics ampicillin (amp), kanamycin (kan), or tetracycline (tet) were added to concentrations of 150, 100, and 20 ug/ml respectively, when needed (Sigma-Aldrich, St. Louis MO). *N*-(3-oxo-hexanoyl)-L-homoserine lactone (oxoC6) was obtained from Sigma-Aldrich and dissolved in ethyl acetate that had been acidified by the addition of 0.1 ml glacial acetic acid per liter (EA) [55]. The stock concentration of oxoC6 was 1 mM and it was used at a final concentration of 1 µM. Solvent controls were performed by using EA alone at 0.1%. All plasmids were prepared from JS198, a r^−^m^+^ *Salmonella*, before electroporation into restriction-proficient *Salmonella* strains [56].

### Construction of *srgE-tnpR-lacZY* fusions

A FRT site was inserted between the stop codons and the transcription terminator of the *srgE* gene using λ Red recombination with PCR primers BA723 (TTGTATGGGGCATATAAAAAGAAATAGTAACATATGAATATCCTCCTTAG) and BA908 (TCAAATGCCAGACTTCCGCTACCAGACGGTGTGTAGGCTGGAGCTGCTTC). The first 30 nucleotides of BA723 bind the end of the *srgE* gene (nt 15344-15315 of Genbank accession number AE008767), pointing downstream, and ending with the two stop codons which are underlined. (The *srgE* gene has two adjacent stop codons.) The next 20 nucleotides match the Priming Site 2 sequence of Datsenko and Wanner's mutagenesis system [57]. The first 30 nucleotides of BA908 bind downstream of the *srgE* gene (nt 15275-15304 of Genbank accession number AE008767), pointing upstream. The next 20 nucleotides match the Priming Site 1 sequence of Datsenko and Wanner's mutagenesis system [57]. The insertion of the antibiotic resistance marker was verified using PCR with two different primer sets, each containing a primer that binds within the antibiotic resistance gene and a second that binds either upstream or downstream of the intended insertion site. The antibiotic resistance cassette was then removed using the temperature sensitive plasmid pCP20 carrying the FLP recombinase [58]. Kanamycin susceptible strains containing pCP20 were electroporated with the pCE71, pCE73, and pCE75 suicide plasmids followed by selection on LB kan plates at 37°C. These suicide plasmids encode a FRT site followed by a promoterless *tnpR-lacZY* [37]. A Flp-mediated site-specific recombination recombines the FRT site on the suicide vector with the *srgE10*-FRT site in the chromosome resulting in integration of the vector into the chromosome [56]. Growth of the resulting colonies at 37°C was sufficient to cure pCP20 and this was confirmed by screening for ampicillin sensitivity. Integration of the *tnpR-lacZY* fusion plasmid into the *srgE10*-FRT site was confirmed using PCR. Once the *srgE10*-*tnpR-lacZY* fusion strains were confirmed, an *sdiA1*::mTn*3* mutation was introduced from BA612 by transduction using P22HTint [59].

### Animals

Female CBA/J mice (8–10 weeks old) were obtained from Jackson Laboratories (Bar Harbor, ME). Female Hartley albino guinea pigs (retired breeders) and female New Zealand albino Rabbits (250 g) were obtained from Charles River Laboratories (Wilmington, MA). All animals were caged separately. Red-eared slider turtles (*Trachemys scripta elegans*) of mixed sex were obtained from Concordia Turtle Farms (Wildsville, LA). Turtles were kept in individual cages containing water and a basking support. The cages, water, and basking supports were sterilized and changed daily. All experiments performed with mice, guinea pigs, rabbits, and turtles were performed at Ohio State University with IACUC approved protocol #2006A0037.

Six domestic female pigs (30–35 pounds) were obtained from North Carolina State University Farms. Pigs were housed three to a stall and food and water were provided ad libitum. This experiment was performed at North Carolina State University with IACUC approved protocol #02-018-B.

Three Holstein calves, 2 male and 1 female, were obtained from Texas A&M University Farms. Calves were three to four months old and weighed from 190–300 lbs. Calves were housed together and food (a pelleted feed that was 12% protein and 3% fat that is primarily comprised of corn and cottonseed meal) and water were provided ad libitum. Calves were also allowed to graze. The calf experiment was performed at Texas A&M University with AUP approved protocol #2003-178.

Chicks were obtained as Fertilized SPAFAS Specific pathogen-free layer eggs (Spafas, Inc., (Roanoke, IL)). The hatchlings were housed in a single Horsefall isolator with 45–50% humidity under constant light at 35°C. Food and water were provided ad libitum. *Salmonella* infections were performed one week after hatching at Washington University under Animal Welfare Assurance number A3381-01.

All animals (except the certified specific pathogen-free chicks) were pre-tested for the presence of kanamycin resistant *Salmonella* by serially diluting and plating fecal samples onto XLD-kan. No animals were positive for kanamycin resistant *Salmonella* after overnight incubation, and no other microbes grew on the plates for several days.

### Measurement of resolvase activity in vitro

#### Liquid broth assay

Wild-type or *sdiA* mutant RIVET strains were inoculated in triplicate from glycerol stocks into 5 ml of LB kan broth containing either 1 µM oxoC6 or the solvent control 0.1% EA and incubated overnight with shaking at 37°C. The resulting cultures were then serially diluted onto LB kan plates. Isolated colonies were screened for tetracycline resistance.

#### Motility agar assay

Wild-type or *sdiA* mutant RIVET strains were stabbed into LB motility agar containing 1 µM oxoC6 or the solvent control 0.1% EA and incubated overnight at 37°C. Colonies were recovered by stabbing the motility agar with a sterile wooden inoculating stick and streaking to isolation on LB kan plates. Isolated colonies were screened for tetracycline resistance.

#### Solid agar assay

Wild-type or *sdiA* mutant RIVET strains were struck to isolation on LB kan plates containing 1 µM oxoC6 or 0.1% EA and incubated overnight at 37°C. Isolated colonies were screened for tetracycline resistance.

### Measurement of resolvase activity in animals

Overnight cultures of the wild-type and *sdiA* mutant RIVET strains (JNS3206 and JNS3226) were grown in LB kan tet at 37°C. The next morning the overnight cultures were centrifuged at 5,000×g and resuspended in fresh LB lacking antibiotics. A 1∶1 mixture of the cultures was used to inoculate the animals intragastrically (200 µl for mice, 100 µl of concentrate for chicks (1 ml resuspended in 100 µl), 4 ml for pigs (1 ml diluted to 4 ml), 10 ml for calves, 1 ml for turtles, guinea pigs, and rabbits. Dilution plating of the inoculum was used to determine the actual dose administered. Screening the isolated colonies for tetracycline resistance indicated that in each experiment no colonies resolved before the beginning of the experiment. The percentage of wild-type *Salmonella* in each inoculum was determined by screening the colonies for ampicillin resistance with the wild-type being amp sensitive and the *sdiA1*::mTn*3* mutant being amp resistant.

For most animals, fresh fecal samples were collected at time points and resuspended in phosphate-buffered saline (PBS) followed by serial dilution and plating on XLD-kan agar. Isolated colonies were then screened for ampicillin and tetracycline resistance. For the turtle experiments, cloacal swabs were collected, vortexed in 1 ml PBS, and plated on XLD-kan. For pigs, a Jorvet fecal sampling loop (Livestock Concepts) was inserted into the rectum, withdrawn, and the feces removed by agitating the loop in a 15 ml conical tube containing 1 ml PBS. Chicks were sampled in groups of three each day post-infection for a total of four days. They were euthanized and fecal samples were collected directly from the cecum and distal large intestine.

### Clearance of *Aeromonas* from turtles

In an attempt to eradicate *Aeromonas* from turtles, the following four steps were performed every 48 hours for two weeks. First, a cloacal swab from each turtle was collected and tested for the presence of *Aeromonas* and *Salmonella* by plating on XLD, LB, and blood agar. Second, the turtles received an IM injection in the front leg of the antibiotic Amikacin at a dose of 5 mg/kg. The injections were performed in alternating legs. Third, immediately after the injection the turtle was bathed in 0.2% chlorhexidine gluconate (except around the eyes) and rinsed with sterile water to remove any external *Aeromonas* or *Salmonella* from the animal. Fourth, the animal was placed into a new sterile container containing sterile water. On the days when no injections were performed the turtles were moved to a new sterile container containing sterile water.

## References

[1] Ahmer BM (2004). Cell-to-cell signalling in *Escherichia coli* and *Salmonella enterica*.. Mol Microbiol.

[2] Smith JN, Ahmer BM (2003). Detection of other microbial species by *Salmonella*: expression of the SdiA regulon.. J Bacteriol.

[3] Michael B, Smith JN, Swift S, Heffron F, Ahmer BM (2001). SdiA of *Salmonella enterica* is a LuxR homolog that detects mixed microbial communities.. J Bacteriol.

[4] Ahmer BMM, Reeuwijk Jv, Timmers CD, Valentine PJ, Heffron F (1998). *Salmonella typhimurium* encodes an SdiA homolog, a putative quorum sensor of the LuxR family, that regulates genes on the virulence plasmid.. Journal of Bacteriology.

[5] Fuqua C, Winans SC, Greenberg EP (1996). Census and consensus in bacterial ecosystems: the LuxR-LuxI family of quorum sensing transcriptional regulators.. Annual Reviews of Microbiology.

[6] Greenberg EP (2003). Bacterial communication and group behavior.. J Clin Invest.

[7] Waters CM, Bassler BL (2005). Quorum sensing: cell-to-cell communication in bacteria.. Annu Rev Cell Dev Biol.

[8] Visick KL, Foster J, Doino J, McFall-Ngai M, Ruby EG (2000). Vibrio fischeri lux genes play an important role in colonization and development of the host light organ.. J Bacteriol.

[9] Visick KL, McFall-Ngai MJ (2000). An exclusive contract: specificity in the *Vibrio fischeri-Euprymna scolopes* partnership.. J Bacteriol.

[10] Lupp C, Urbanowski M, Greenberg EP, Ruby EG (2003). The *Vibrio fischeri* quorum-sensing systems ain and lux sequentially induce luminescence gene expression and are important for persistence in the squid host.. Mol Microbiol.

[11] Swift S, Williams P, Stewart GSAB, Dunny GM, Winans S (1999). *N*-acylhomoserine lactones and quorum sensing in proteobacteria.. Cell-Cell Signalling in Bacteria.

[12] Smith D, Wang JH, Swatton JE, Davenport P, Price B (2006). Variations on a theme: diverse N-acyl homoserine lactone-mediated quorum sensing mechanisms in gram-negative bacteria.. Sci Prog.

[13] Bassler BL, Wright M, Showalter RE, Silverman MR (1993). Intercellular signalling in *Vibrio harveyi*: sequence and function of genes regulating expression of luminescence.. Molecular Microbiology.

[14] Gilson L, Kuo A, Dunlap PV (1995). AinS and a new family of autoinducer synthesis proteins.. Journal of Bacteriology.

[15] Hanzelka BL, Greenberg EP (1996). Quorum sensing in *Vibrio fischeri*: evidence that *S*-adenosylmethionine is the amino acid substrate for autoinducer synthesis.. Journal of Bacteriology.

[16] More MI, Finger LD, Stryker JL, Fuqua C, Eberhard A (1996). Enzymatic synthesis of a quorum-sensing autoinducer through use of defined substrates.. Science.

[17] Schaefer AL, Val DL, Hanzelka BL, Cronan JE, Greenberg EP (1996). Generation of cell-to-cell signals in quorum sensing: acyl homoserine lactone synthase activity of a purified *Vibrio fischeri* LuxI protein.. Proceedings of the National Academy of Sciences of the United States of America.

[18] Milton DL, Chalker VJ, Kirke D, Hardman A, Camara M (2001). The LuxM homologue VanM from Vibrio anguillarum directs the synthesis of N-(3-hydroxyhexanoyl)homoserine lactone and N-hexanoylhomoserine lactone.. J Bacteriol.

[19] Fuqua C, Eberhard A, Dunny GM, Winans SC (1999). Signal generation in autoinduction systems: synthesis of acylated homoserine lactones by LuxI-type proteins.. Cell-Cell Signaling in Bacteria.

[20] Steindler L, Venturi V (2007). Detection of quorum-sensing N-acyl homoserine lactone signal molecules by bacterial biosensors.. FEMS Microbiol Lett.

[21] Janssens JC, Metzger K, Daniels R, Ptacek D, Verhoeven T (2007). Synthesis of N-acyl homoserine lactone analogues reveals strong activators of SdiA, the Salmonella enterica serovar Typhimurium LuxR homologue.. Appl Environ Microbiol.

[22] Yao Y, Martinez-Yamout MA, Dickerson TJ, Brogan AP, Wright PE (2006). Structure of the Escherichia coli quorum sensing protein SdiA: activation of the folding switch by acyl homoserine lactones.. J Mol Biol.

[23] Friedrich MJ, Kinsey NE, Vila J, Kadner RJ (1993). Nucleotide sequence of a 13.9 kb segment of the 90 kb virulence plasmid of *Salmonella typhimurium*: the presence of fimbrial biosynthetic genes.. Molecular Microbiology.

[24] Baumler AJ, Tsolis RM, Bowe FA, Kusters JG, Hoffmann S (1996). The *pef* fimbrial operon of *Salmonella typhimurium* mediates adhesion to murine small intestine and is necessary for fluid accumulation in the infant mouse.. Infection & Immunity.

[25] Nicholson B, Low D (2000). DNA methylation-dependent regulation of *pef* expression in *Salmonella typhimurium*.. Molecular Microbiology.

[26] Bouwman CW, Kohli M, Killoran A, Touchie GA, Kadner RJ (2003). Characterization of SrgA, a *Salmonella enterica* serovar Typhimurium virulence plasmid-encoded paralogue of the disulfide oxidoreductase DsbA, essential for biogenesis of plasmid-encoded fimbriae.. J Bacteriol.

[27] Lin D, Rao CV, Slauch JM (2008). The *Salmonella* SPI1 type three secretion system responds to periplasmic disulfide bond status via the flagellar apparatus and the RcsCDB system.. J Bacteriol.

[28] Cirillo DM, Heffernan EJ, Wu L, Harwood J, Fierer J (1996). Identification of a domain in Rck, a product of the *Salmonella typhimurium* virulence plasmid, required for both serum resistance and cell invasion.. Infection & Immunity.

[29] Hackett J, Wyk P, Reeves P, Mathan V (1987). Mediation of serum resistance in *Salmonella typhimurium* by an 11- kilodalton polypeptide encoded by the cryptic plasmid.. Journal of Infectious Disease.

[30] Heffernan EJ, Harwood J, Fierer J, Guiney D (1992). The *Salmonella typhimurium* virulence plasmid complement resistance gene *rck* is homologous to a family of virulence-related outer membrane protein genes, including *pagC* and *ail*.. Journal of Bacteriology.

[31] Heffernan EJ, Reed S, Hackett J, Fierer J, Roudier C (1992). Mechanism of resistance to complement-mediated killing of bacteria encoded by the *Salmonella typhimurium* virulence plasmid gene *rck*.. Journal of Clinical Investigation.

[32] Crago AM, Koronakis V (1999). Binding of extracellular matrix laminin to *Escherichia coli* expressing the *Salmonella* outer membrane proteins Rck and PagC.. FEMS Microbiology Letters.

[33] Backhed F, Ley RE, Sonnenburg JL, Peterson DA, Gordon JI (2005). Host-bacterial mutualism in the human intestine.. Science.

[34] Camilli A, Beattie DT, Mekalanos JJ (1994). Use of genetic recombination as a reporter of gene expression.. Proc Natl Acad Sci U S A.

[35] Camilli A, Mekalanos JJ (1995). Use of recombinase gene fusions to identify *Vibrio cholerae* genes induced during infection.. Molecular Microbiology.

[36] Slauch JM, Camilli A (2000). IVET and RIVET: use of gene fusions to identify bacterial virulence factors specifically induced in host tissues.. Methods Enzymol.

[37] Merighi M, Ellermeier CD, Slauch JM, Gunn JS (2005). Resolvase-in vivo expression technology analysis of the *Salmonella enterica* serovar Typhimurium PhoP and PmrA regulons in BALB/c mice.. J Bacteriol.

[38] Lee SH, Hava DL, Waldor MK, Camilli A (1999). Regulation and temporal expression patterns of *Vibrio cholerae* virulence genes during infection.. Cell.

[39] Kingsley RA, Humphries AD, Weening EH, De Zoete MR, Winter S (2003). Molecular and phenotypic analysis of the CS54 island of Salmonella enterica serotype typhimurium: identification of intestinal colonization and persistence determinants.. Infect Immun.

[40] Erickson DL, Nsereko VL, Morgavi DP, Selinger LB, Rode LM (2002). Evidence of quorum sensing in the rumen ecosystem: detection of N-acyl homoserine lactone autoinducers in ruminal contents.. Can J Microbiol.

[41] Roche DM, Byers JT, Smith DS, Glansdorp FG, Spring DR (2004). Communications blackout? Do N-acylhomoserine-lactone-degrading enzymes have any role in quorum sensing?. Microbiology.

[42] Wang YJ, Leadbetter JR (2005). Rapid acyl-homoserine lactone quorum signal biodegradation in diverse soils.. Appl Environ Microbiol.

[43] Dong YH, Zhang LH (2005). Quorum sensing and quorum-quenching enzymes.. J Microbiol.

[44] McGarey DJ, Milanesi L, Foley DP, Reyes B, Frye LC (1991). The role of motile aeromonads in the fish disease, ulcerative disease syndrome (UDS).. Experientia.

[45] Cipriano RC (2001). Aeromonas hydrophila and motile aeromonad septicemias of fish. Fish Disease Leaflet 68..

[46] Kozinska A (2007). Dominant pathogenic species of mesophilic aeromonads isolated from diseased and healthy fish cultured in Poland.. J Fish Dis.

[47] Sen K, Lye D (2007). Importance of flagella and enterotoxins for Aeromonas virulence in a mouse model.. Can J Microbiol.

[48] Kirke DF, Swift S, Lynch MJ, Williams P (2004). The *Aeromonas hydrophila* LuxR homologue AhyR regulates the *N*-acyl homoserine lactone synthase, AhyI positively and negatively in a growth phase-dependent manner.. FEMS Microbiol Lett.

[49] Swift S, Karlyshev AV, Fish L, Durant EL, Winson MK (1997). Quorum sensing in *Aeromonas hydrophila* and *Aeromonas salmonicida*: identification of the LuxRI homologs AhyRI and AsaRI and their cognate N-acylhomoserine lactone signal molecules.. Journal of Bacteriology.

[50] Swift S, Lynch MJ, Fish L, Kirke DF, Tomas JM (1999). Quorum sensing-dependent regulation and blockade of exoprotease production in *Aeromonas hydrophila*.. Infect Immun.

[51] Kodjo A, Villard L, Prave M, Ray S, Grezel D (1997). Isolation and identification of Salmonella species from chelonians using combined selective media, serotyping and ribotyping.. Zentralbl Veterinarmed B.

[52] Pasmans F, De Herdt P, Haesebrouck F (2002). Presence of Salmonella infections in freshwater turtles.. Vet Rec.

[53] Shane SM, Gilbert R, Harrington KS (1990). Salmonella colonization in commercial pet turtles (Pseudemys scripta elegans).. Epidemiol Infect.

[54] CDC CfDC (1999). Salmonella surveillance: annual tabulation summary, 1998.

[55] Pearson JP, Gray KM, Passador L, Tucker KD, Eberhard A (1994). Structure of the autoinducer required for expression of *Pseudomonas aeruginosa* virulence genes.. Proceedings of the National Academy of Sciences of the United States of America.

[56] Ellermeier CD, Janakiraman A, Slauch JM (2002). Construction of targeted single copy *lac* fusions using lambda Red and FLP-mediated site-specific recombination in bacteria.. Gene.

[57] Datsenko KA, Wanner BL (2000). One-step inactivation of chromosomal genes in *Escherichia coli* K-12 using PCR products.. Proc Natl Acad Sci U S A.

[58] Cherepanov PP, Wackernagel W (1995). Gene disruption in *Escherichia coli*: TcR and KmR cassettes with the option of Flp-catalyzed excision of the antibiotic-resistance determinant.. Gene.

[59] Maloy SR, Stewart VJ, Taylor RK (1996). Genetic Analysis of Pathogenic Bacteria: A Laboratory Manual..

[60] Simon R, Priefer U, Puhler A (1983). A broad-host range mobilization system for in vivo genetic engineering: transposon mutagenesis in gram-negative bacteria.. Bio/Technology.

[61] Hill CW, Harnish BW (1981). Inversions between ribosomal RNA genes of *Escherichia coli*.. Proc Natl Acad Sci U S A.

